# Curcumin Protects Neonatal Rat Cardiomyocytes against High Glucose-Induced Apoptosis via PI3K/Akt Signalling Pathway

**DOI:** 10.1155/2016/4158591

**Published:** 2016-02-16

**Authors:** Wei Yu, Wenliang Zha, Zhiqiang Ke, Qing Min, Cairong Li, Huirong Sun, Chao Liu

**Affiliations:** ^1^Hubei Province Key Laboratory on Cardiovascular, Cerebrovascular, and Metabolic Disorders, Hubei University of Science and Technology, Xianning 437100, China; ^2^Department of Pharmacology, Hubei University of Science and Technology, Xianning 437100, China; ^3^Department of Cardiology, Zhongnan Hospital of Wuhan University, Wuhan 430071, China

## Abstract

The function of curcumin on NADPH oxidase-related ROS production and cardiac apoptosis, together with the modulation of protein signalling pathways, was investigated in cardiomyocytes. Primary cultures of neonatal rat cardiomyocytes were exposed to 30 mmol/L high glucose with or without curcumin. Cell viability, apoptosis, superoxide formation, the expression of NADPH oxidase subunits, and potential regulatory molecules, Akt and GSK-3*β*, were assessed in cardiomyocytes. Cardiomyocytes exposure to high glucose led to an increase in both cell apoptosis and intracellular ROS levels, which were strongly prevented by curcumin treatment (10 *μ*M). In addition, treatment with curcumin remarkably suppressed the increased activity of Rac1, as well as the enhanced expression of gp91^phox^ and p47^phox^ induced by high glucose. Lipid peroxidation and SOD were reversed in the presence of curcumin. Furthermore, curcumin treatment markedly inhibited the reduced Bcl-2/Bax ratio elicited by high glucose exposure. Moreover, curcumin significantly increased Akt and GSK-3*β* phosphorylation in cardiomyocytes treated with high glucose. In addition, LY294002 blocked the effects of curcumin on cardiomyocytes exposure to high glucose. In conclusion, these results demonstrated that curcumin attenuated high glucose-induced cardiomyocyte apoptosis by inhibiting NADPH-mediated oxidative stress and this protective effect is most likely mediated by PI3K/Akt-related signalling pathway.

## 1. Introduction

Diabetes mellitus (DM) is becoming a global health problem that is afflicting millions of people. According to the investigation conducted by the International Diabetes Federation (IDF), the incidence of DM is rapidly increasing and the total number of people with DM will reach 592 million in 2035 [1]. Studies have indicated that diabetic people have a 2- to 5-fold increased risk of developing heart failure [2] and that more than 50%–80% of diabetic patients die from diabetic cardiovascular complications [3]. Diabetic cardiomyopathy (DCM), as a major complication of DM, was initially proposed by Rubler in 1972 [4]. DCM is characterized by structural and functional cardiac disorder occurring independently of coronary artery disease and hypertension [5]. Although many research studies have attempted to elucidate its underlying mechanisms, the aetiology of DCM has never been directly determined. Numerous studies utilizing experimental animal models and clinical diabetes patients reported that diabetes enhances cardiomyocyte apoptosis not only simply in animals but also in patients [6, 7]. Thus, cell death by apoptosis likely plays an important role in triggering the pathogenic changes in DCM [8]. Cardiomyocyte apoptosis can cause a loss of cardiac contractile muscle tissue, which eventually leads to left ventricular remodeling [9].

Both type 1 and type 2 DM are associated with long-standing hyperglycemia. Chronic hyperglycemia has been shown to directly participate in the pathogenesis of DM-induced cardiac injury by promoting excessive oxidative stress in the heart [10], which increases cardiomyocyte apoptosis in both human and experimental DCM. Overproduction of reactive oxygen species (ROS) and a diminished antioxidant defence system are linked to enhanced oxidative stress in the heart in DM. Consequently, if the balance between ROS generation and ROS scavenging systems is broken, superoxide accumulates and results in cellular damage or dysfunction. Given the injurious effects of ROS in DCM, increasing attention has been placed on the administration of antioxidant agents as a compensatory therapeutic approach in DCM [11].

Curcumin, a major constituent derived from the root of* Curcuma longa*, has been used as a spice and food additive in India since ancient times. Today, interest in curcumin has grown rapidly due to its diverse array of biological and pharmacological activities, and it was shown to have the potential to treat inflammatory and cardiovascular diseases and cancer [12]. Curcumin has antioxidant properties that are responsible for its cardioprotective effect by enhancing antioxidant defences and eradicating ROS [13]. Additional studies have indicated that C66, a curcumin analogue, has a protective role against high glucose-induced cardiac damage via inactivation of the JNK pathway [14]. Our early stage study demonstrated that curcumin reduces cardiomyocyte remodeling and improves cardiac dysfunction by inhibiting inappropriate apoptosis in diabetic rats [15], but the mechanism through which curcumin inhibits cardiomyocyte apoptosis and oxidative stress remains unknown.

Therefore, this study was performed to determine the action of curcumin against high glucose-induced cardiac injury and elucidate the molecular mechanism of cardiomyocyte protection by exposing primary neonatal rat cardiomyocytes to a high concentration of glucose.

## 2. Materials and Methods

### 2.1. Animals

One- to three-day-old Sprague-Dawley rats were obtained from the experimental animal centre at Hubei University of Science and Technology. The Committee of Experimental Animals of Hubei University of Science and Technology approved this study. All animals used in this study were cared for and experimented on in accordance with the recommendations in the Guide for the Care and Use of Laboratory Animals published by the National Institutes of Health.

### 2.2. Primary Culture of Neonatal Rat Cardiomyocytes and Treatment

Neonatal rat cardiomyocytes were isolated as previously described with slight modifications [16]. The cardiomyocytes were cultured in DMEM containing 10% FBS (Gibco Life of Cells, USA), 100 U/mL penicillin, and 100 mg/mL streptomycin in a humidified air containing 5% CO_2_ at 37°C. When the cardiomyocytes reached 70%–80% confluence, the cells were randomized into the experimental groups: 5.5 mmol/L D-glucose as the normal (NG) group, 30 mmol/L D-glucose (Sigma, USA) as the high glucose (HG) group, or identical concentrations of mannitol as an osmotic control group containing 5.5 mmol/L D-glucose plus 24.5 mmol/L mannitol for 24 h in the presence or absence of curcumin (10 *μ*mol/L). A subset of cardiomyocytes were exposed to LY294002 for 1 h before administration of high glucose and curcumin.

### 2.3. Assessment of Cell Viability

Cell viability was assessed with a Cell Counting Kit-8 assay kit (CCK-8, Dojindo Molecular Technologies, Japan) in 96-well plates following the instructions from the manufacturer.

### 2.4. Analysis of Biochemical Parameters

Lactate dehydrogenase (LDH) and aspartate amino transferase (AST) released into the culture medium as well as the malondialdehyde (MDA) level and superoxide dismutase (SOD) activity in cells were determined using the associated enzyme activity assay kits (Nanjing Jiancheng Bioengineering Research Institute, China).

### 2.5. Intracellular ROS Measurement

Intracellular superoxide anions were examined using the fluorescence probe dihydroethidium (DHE). Cardiomyocytes were cultured in a dark chamber at 37°C for 30 min after the application of 10 *μ*mol/L DHE (Life Technology, USA) and were washed twice with PBS. Images of the cardiomyocytes were captured and analysed immediately under an inverted fluorescence microscopy (Olympus IX71, Japan).

Intracellular ROS accumulation was assessed by DCFH-DA staining (Beyotime, China). Cultured cells were incubated in DMEM with 10 *μ*mol/L DCFH-DA at 37°C for 30 min. ROS production was detected by a Bio-Tek fluorometric imaging plate reader (excitation at 485 nm and emission at 528 nm).

Furthermore, intracellular ROS was also measured by high performance liquid chromatography (HPLC) (SHIMADZU, LC-20AD, Japan) using a DHE fluorescent probe as previously described [17]. Briefly, cardiomyocytes were treated with DHE (10 *μ*mol/L) for 30 min and then incubated with 0.1% Triton X-100 dissolved in PBS to permeabilize the cell membrane. Protein determination was performed using a Bicinchoninic Acid (BCA) protein assay kit (Beyotime, China). Then, 100 *μ*L of cell lysate was added to an equal volume of 0.2 mol/L HClO_4_ in methanol, and the mixture was put on ice for 2 h to precipitate the proteins. Afterwards, the resulting mixture was centrifuged at 20,000 g at 4°C for 30 min. Then the supernatant was collected and neutralized with 1 mol/L potassium phosphate buffer (pH 2.6). The supernatant was spun again for 15 min and subjected to the HPLC analysis (excitation at 490 nm and emission at 596 nm).

### 2.6. TUNEL Assay

Apoptotic cardiomyocytes were detected using a terminal deoxynucleotidyl transferase dUTP nick end labelling (TUNEL) assay kit obtained from Roche Applied Science. Briefly, 4% paraformaldehyde and 0.1% Triton X-100 were used to fix and permeabilize the cardiomyocytes on glass slides. After rinsing the cardiomyocytes with PBS, they were added and incubated with the TUNEL reagents according to the instructions from the manufacturer. Images were captured under a fluorescence microscope (Olympus BX53, Japan) and the proportion of TUNEL-positive cells was estimated using the following formula: TUNEL-positive cardiomyocytes/total number of cardiomyocytes × 100%.

### 2.7. Flow Cytometry

Flow cytometry was performed using an Annexin V-FITC Apoptosis Detection Kit (Best Bio, China) following the manufacturer's protocol. Briefly, after being treated with the appropriate drugs, the cardiomyocytes were harvested with trypsin and washed with cold PBS. Afterwards, the collected cardiomyocytes were isolated by centrifugation, resuspended in 500 *μ*L of 1x binding buffer, and treated with 5 *μ*L Annexin V-FITC and 5 *μ*L PI for 15 min at 4°C in the dark. Apoptotic cardiomyocytes were detected by flow cytometer (Becton Dickinson, USA).

### 2.8. Determination of Rac1 Activity

Rac1 activity was determined using a Rac1 Activation Assay Kit (Millipore, USA) following the instructions from the manufacturer. Cultured cardiomyocytes were homogenized with 1x MLB. Rac-GTP was immunoprecipitated using PAK1-PBD agarose beads coated with an anti-active Rac-GTP mouse monoclonal antibody. Finally, the extracts were analysed by western blot.

### 2.9. Western Blot Analysis

Cardiomyocytes were lysed with 1x RIPA lysis buffer (Cell Signalling Technology, USA). After centrifugation, the lysates were clarified, and the supernatants fractions were isolated. Protein concentrations in cells were defined by the BCA protein assay. Approximately 30–50 *μ*g of protein was loaded and separated by SDS-PAGE gels and then transferred to a PVDF membrane. After blocking the membrane with 5% nonfat milk, the following primary antibodies were used for western blot: Bcl-2, Bax, Akt, GSK-3*β*, phospho-Akt (Ser473), phospho-GSK-3*β* (Ser9) (Cell Signalling Technology, USA), gp91^phox^, p47^phox^, and *β*-actin (Santa Cruz Biotechnology, USA). Then the membrane was probed with appropriate secondary antibodies. Finally, the blots were visualized using a chemiluminescence system (Pierce Biosciences, USA). Image analysis software (GeneTools from SynGene) was used to quantify the immunoblots.

### 2.10. Statistics

The values are expressed as mean ± SD from repeated experiments. Statistical analysis was performed using ANOVA, and a *P* value of <0.05 was considered to indicate a significant difference for all the values.

## 3. Results

### 3.1. Curcumin Inhibited High Glucose-Induced Cardiomyocytes Injury

**Figure 1:**
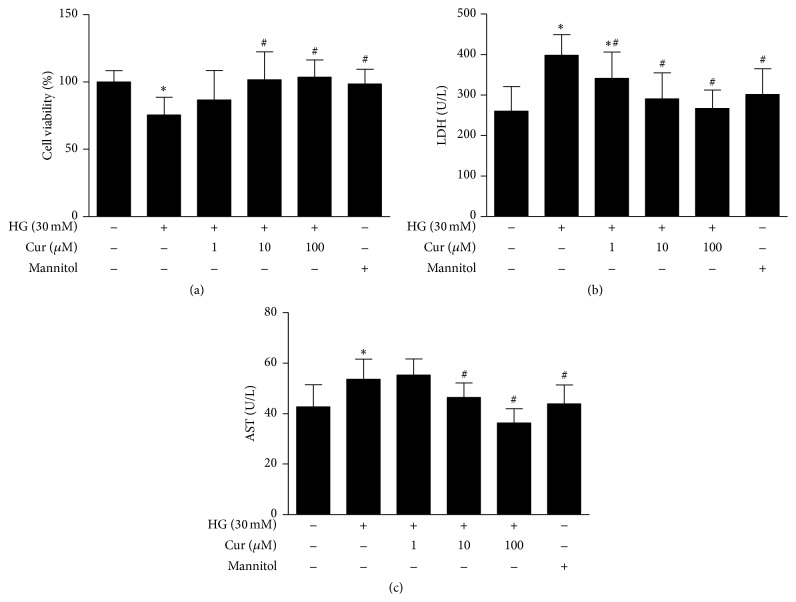
Curcumin increased cell viability and inhibited injury in cardiomyocytes exposure to high glucose. (a) Cell viability was examined with a CCK-8 assay. (b) Curcumin decreased the level of LDH in the supernatant. (c) Curcumin decreased the level of AST in the supernatant. Values are presented as mean ± SD. ^*∗*^ *P* < 0.05 versus NG group. ^#^ *P* < 0.05 versus HG group. *n* = 10.

To ascertain the role of curcumin in cell survival, we examined the viability of primary cultured neonatal rat cardiomyocytes incubated with different doses of curcumin for 24 h using a CCK-8 assay. As presented in Figure 1(a), compared with the NG group, cell viability was markedly decreased at the high glucose concentration of 30 mmol/L, and mannitol (30 mmol/L) employed as an osmotic control agent did not mimic the effects of 30 mmol/L D-glucose. In the cardiomyocytes exposed to high glucose, curcumin treatment increased cell viability in a dose-dependent manner.

LDH and AST are oxidoreductase enzymes that are present in the cytosol of animals and plants. They are highly stable enzymes that can be used to evaluate tissue and cell damage. As shown in Figures 1(b) and 1(c), the amounts of LDH and AST released by the cardiomyocytes were much higher in the HG group than those in the NG group. Interestingly, the cardiomyocytes exposed to high glucose and treated with curcumin released significantly lower amounts of LDH and AST into the medium than the cardiomyocytes undergoing high glucose alone.

### 3.2. Curcumin Abrogated High Glucose-Induced Cardiomyocytes Apoptosis

**Figure 2:**
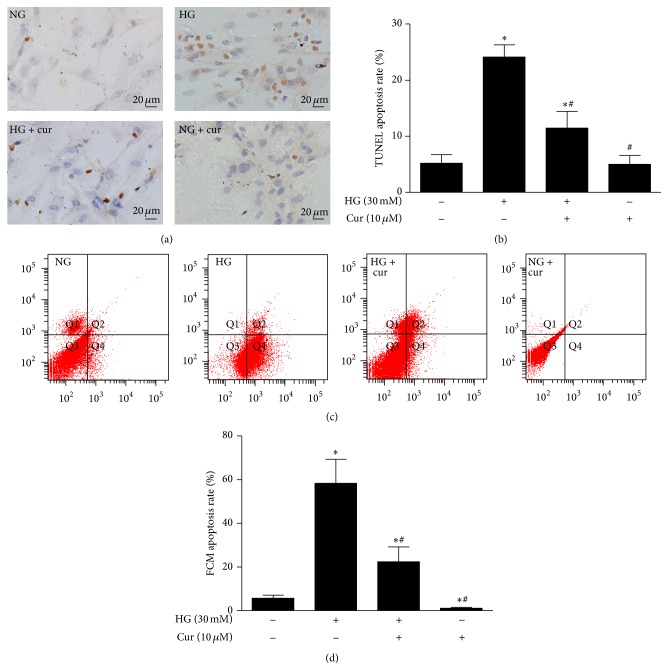
Curcumin inhibited high glucose-induced cardiomyocytes apoptosis. (a) Representative images of apoptotic cardiomyocytes stained by TUNEL (magnification = 400x, bar is 20 *μ*m). (b) Quantitative analysis of TUNEL staining. (c) Representative images of apoptotic cardiomyocytes observed using FCM. (d) Quantitative analysis of the FCM results. Values are presented as mean ± SD. ^*∗*^ *P* < 0.05 versus NG group. ^#^ *P* < 0.05 versus HG group.

Apoptotic cardiomyocytes were detected by both TUNEL staining and flow cytometry. The TUNEL assay showed few apoptotic cardiomyocytes in the NG group and a greater number of apoptotic cardiomyocytes in the HG group. However, cotreatment with high glucose and curcumin (10 *μ*M) abrogated the increase in TUNEL-positive cells triggered by high glucose (Figures 2(a) and 2(b)).

The flow cytometric analysis also suggested that the administration of curcumin resulted in an evident decrease in the number of apoptotic bodies compared to cardiomyocytes exposed to high glucose that were not treated with curcumin (Figures 2(c) and 2(d)).

**Figure 3:**
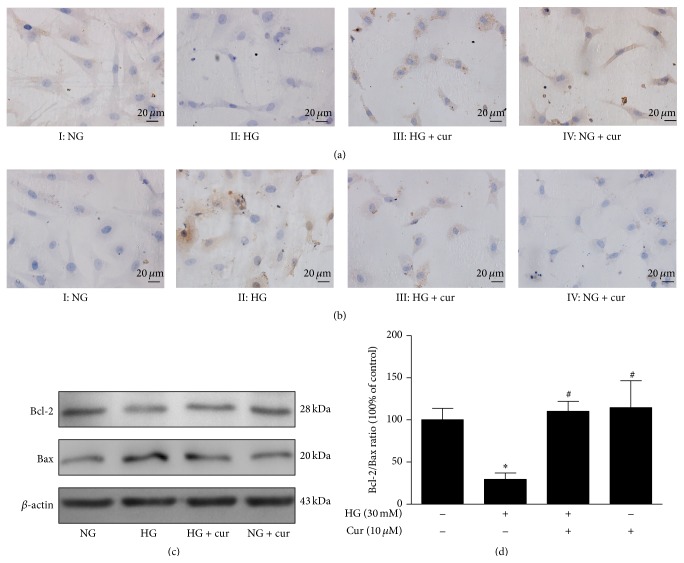
Curcumin regulated the expression of Bax and Bcl-2 in cardiomyocytes exposed to high glucose. (a) Representative images of Bcl-2 by immunohistochemical staining (magnification = 400x, bar is 20 *μ*m). (b) Representative images of Bax by immunohistochemical staining (magnification = 400x, bar is 20 *μ*m). (c) Representative images of Bax and Bcl-2 expression by western blot. (d) Quantitative analysis of the Bcl-2/Bax ratio. *n* = 3. Values are presented as mean ± SD. ^*∗*^ *P* < 0.05 versus NG group. ^#^ *P* < 0.05 versus HG group.

It is well known that apoptotic-related proteins regulate the progression of apoptosis. Thus, we performed further experiments to investigate if these regulatory proteins contributed to the effect of the inhibition of high glucose-induced apoptosis by curcumin. As indicated by immunohistochemical staining (Figures 3(a) and 3(b)), in contrast to the NG group, the HG group had reduced Bcl-2 expression and enhanced Bax expression. As we predicted, curcumin significantly enhanced Bcl-2 expression and reduced Bax expression in cardiomyocytes exposed to high glucose. Western blot showed that the Bcl-2/Bax ratio was remarkably reduced in the HG group compared to the NG group (Figures 3(c) and 3(d)). After treatment with curcumin, Bcl-2 expression was elevated, Bax expression was greatly reduced, and the Bcl-2/Bax ratio was significantly upregulated.

### 3.3. Curcumin Decreased High Glucose-Induced ROS Generation, Reduced MDA Content, and Increased SOD Activity in Cardiomyocytes

**Figure 4:**
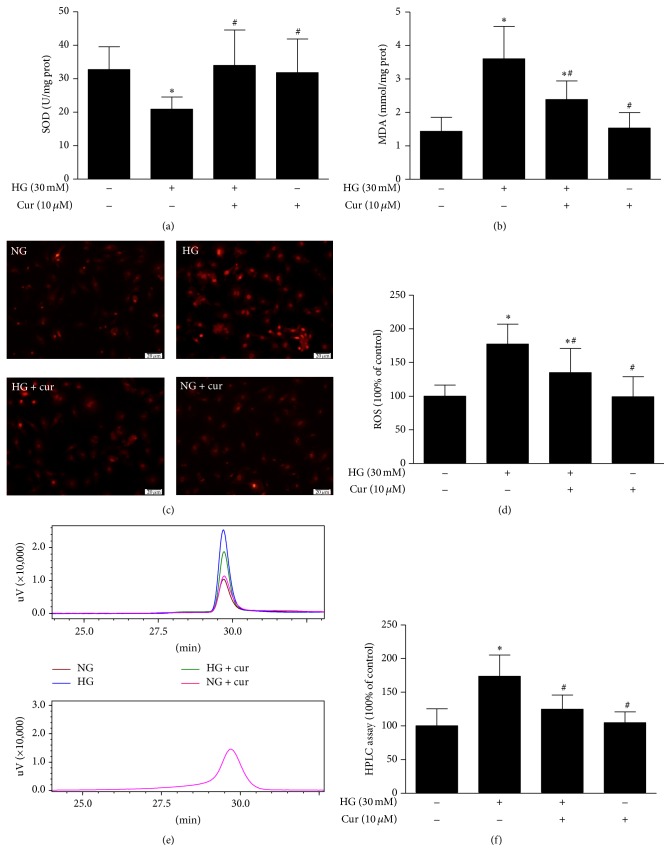
Curcumin suppressed high glucose-induced cardiomyocytes oxidative stress. (a) Curcumin enhanced SOD activity in cardiomyocytes (*n* = 12). (b) Curcumin reduced MDA level in cardiomyocytes (*n* = 10). (c) Representative images of DHE staining (*n* = 3). (d) Quantification of DCFH-DA staining (*n* = 11-12). (e) Representative images of HPLC assay (*n* = 4–6). (f) Quantification of HPLC assay. Values are presented as mean ± SD. ^*∗*^ *P* < 0.05 versus NG group. ^#^ *P* < 0.05 versus HG group.

MDA formation and SOD activity, indexes of lipid superoxide, and oxygen free radical levels were measured in the cardiomyocytes. The MDA level in cardiomyocytes was significantly increased in the HG group. In contrast, SOD activity was found to be decreased when comparing the HG group with the NG group. The cardiomyocytes exposed to high glucose with curcumin markedly decreased the MDA level and enhanced SOD activity compared to those not treated with curcumin (Figures 4(a) and 4(b)).

ROS (a key executor of oxidative stress) was measured by DHE staining, DCFH-DA staining, and HPLC assay, which indicated that the ROS level in the HG group was much higher than that in the NG group. However, the HG-induced increase in ROS was strongly blocked by treatment with curcumin (Figures 4(c)–4(f)).

### 3.4. Curcumin Attenuated High Glucose-Induced Expression of NADPH Oxidase Isoforms in Cardiomyocytes

**Figure 5:**
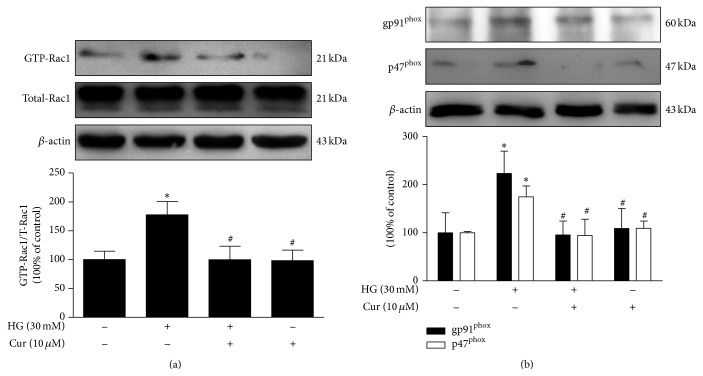
Curcumin decreased high glucose-induced Rac1 activity and the expression of gp91^phox^ and p47^phox^. (a) Rac1 activity assay. (b) Western blots analysis of gp91^phox^ and p47^phox^ expression. *n* = 3. Values are presented as mean ± SD. ^*∗*^ *P* < 0.05 versus NG group. ^#^ *P* < 0.05 versus HG group.

Because NADPH oxidase activation is directly related to increased oxidant production induced by hyperglycemia [18], we evaluated Rac1 activity and gp91^phox^ and p47^phox^ (NADPH subunits) expression. As presented in Figure 5, Rac1 activity in cardiomyocytes was much higher in the HG group than in the NG group. Moreover, increased expression of gp91^phox^ and p47^phox^ was detected in the HG group, while Rac1 activity and the expression of gp91^phox^ and p47^phox^ were markedly inhibited by curcumin in the cardiomyocytes exposed to high glucose. These data suggest that the protective role of curcumin against HG-induced cardiac injury is largely through inhibition of NADPH oxidase-mediated ROS production.

### 3.5. Curcumin Activated the PI3K/Akt/GSK-3*β* Signalling Pathway in Cardiomyocytes

**Figure 6:**
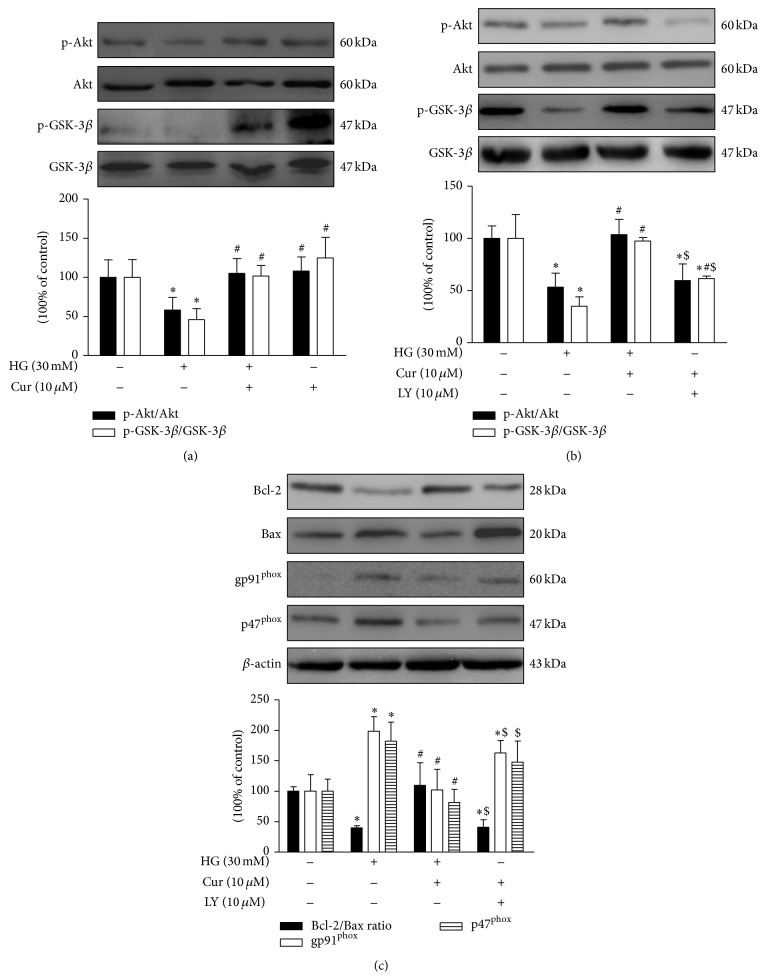
Curcumin inhibited high glucose-induced apoptosis and oxidative stress via activation of Akt in cardiomyocytes. (a) Curcumin upregulated Akt and GSK-3*β* phosphorylation levels. (b) Increased Akt and GSK-3*β* phosphorylation by curcumin were abolished by treatment with LY294002. (c) The effects on the Bcl-2/Bax ratio and the expression of gp91^phox^ and p47^phox^ by curcumin were blocked by treatment with LY294002. *n* = 3. Values are presented as mean ± SD. ^*∗*^ *P* < 0.05 versus NG group. ^#^ *P* < 0.05 versus HG group. ^$^ *P* < 0.05 versus curcumin treatment group.

The activation of the PI3K/Akt signalling pathways is well known to inhibit HG-induced apoptosis [19]. Therefore, we targeted the PI3K/Akt signalling pathway to determine the mechanism through which curcumin inhibits HG-induced apoptosis. Akt and GSK-3*β* phosphorylation were markedly decreased in the HG group as compared with the NG group. Cardiomyocytes treated with curcumin showed a remarkable increase in expression of Akt and GSK-3*β* phosphorylation. Pretreatment with the PI3K inhibitor LY294002 reversed the increased effect of curcumin on Akt and GSK-3*β* phosphorylation. In parallel with that, the expression of Bcl-2 and Bax regulated by curcumin was abolished by treatment with LY294002 and curcumin failed to reduce gp91^phox^ and p47^phox^ expression levels when LY294002 was applied. These results indicate that PI3K/Akt signalling may be involved in the inhibition of apoptosis by curcumin in cardiomyocytes exposed to high glucose (Figure 6).

## 4. Discussion

DCM is frequently seen in asymptomatic diabetic patients. It is now recognized as left ventricular dysfunction associated with increasing the danger of heart failure without hypertension and coronary artery disease or valvular heart diseases [6]. DM-induced left ventricular dysfunction includes impaired systolic and diastolic function, but diastolic dysfunction can occur prior to systolic dysfunction and can be characterized as the early phase of DCM [20]. Several studies have shown a close correlation between left ventricular diastolic dysfunction and myocardial apoptosis and have shown that cardiac function can be improved by sufficient control of myocardial apoptosis [21]. Massive loss of cardiomyocytes due to various apoptotic stimuli occurs, resulting in fibrosis and, eventually, heart failure due to the lack of cardiomyocyte proliferation greatly limiting the generation of new cardiomyocyte. In this context, a clear reduction in cardiomyocyte apoptosis is regarded as a latent therapeutic strategy for the treatment of DCM. In the current work, we found that curcumin lessened cardiomyocyte apoptosis induced by high glucose and observed a curcumin-induced reduction in Bax, which plays a crucial role in mitochondrion-mediated apoptosis by being inserted into the mitochondrial outer membrane and resulting in the release of proapoptotic factors. In contrast, Bcl-2 is an antiapoptotic protein that prevents Bax oligomerization and was increased by curcumin treatment. Thus, curcumin's cardioprotective effects are possibly mediated by normalization of the Bcl-2/Bax ratio.

Cumulative evidence suggests that both cardiomyocyte apoptosis and oxidative stress contribute to the pathogenesis and development of diabetic cardiovascular complications [22]. Previous researches have provided direct evidence that the sustained generation of ROS during oxidative stress leads to cardiomyocyte apoptosis, which contributes to the development of DCM [23]. Emerging evidence has confirmed that activation of NADPH oxidase-generated ROS signalling is related to apoptosis in cardiomyocytes exposed to a hyperglycemic environment [24]. NADPH oxidase contains two membrane-bound subunits (gp91^phox^ and p22^phox^) and four cytosolic regulatory subunits, including p40^phox^, p47^phox^, p67^phox^, and Rac1. Rac1 plays a crucial role in the assembly of NADPH oxidase, which generates superoxide [25] and is a central factor in NADPH-mediated cardiomyocytes apoptosis in response to high glucose levels [26]. Therefore, the deleterious consequences of overactivation of NADPH oxidase in the form of diabetic cardiovascular complications have been well established. NADPH oxidase can impair the redox balance, thus inducing or exacerbating intracellular oxidative stress and resulting in abnormal ROS production. Therefore, inhibition of excessive ROS produced by NADPH oxidase appears to be another possible target for preventing the development of DCM [27]. Consistent with previous studies, we also found that lipid peroxidation levels were enhanced when the cardiomyocytes were exposed to high glucose, which was accompanied by an elevation in ROS generation as a result of the activation of NADPH oxidase. High glucose triggers NADPH oxidase activation by improving Rac1 activation and enhancing gp91^phox^ and gp47^phox^ expression. In agreement with earlier studies that showed that curcumin has a multitude of cardioprotective effects attributed to its efficient antioxidant capacity, we found that treatment with curcumin suppressed a hyperglycemia-induced rise in ROS generation through inactivation of NADPH oxidase.

Impaired Akt/GSK-3*β* signalling pathway has been shown to be involved in the development of metabolic disorders. Akt is responsible for the modulation of cardiovascular functions linked with cardiac growth and survival, contractile function, and coronary angiogenesis [28]. In particular, Akt1 has been demonstrated to play an antagonist role against pathological cardiac hypertrophy, which is an inevitable precursor of heart failure. Consistent with these observations, Akt1^−/−^ mice displayed multiple heart defects, including enhanced cardiac growth and insufficient cardiomyocyte contractility [29]. Furthermore, Akt2 exerts a particularly important impact on the regulation of glucose metabolism and supports cell survival by restraining apoptosis via activation or inactivation of a number of target proteins involved in the process of apoptotic cascades [30, 31]. GSK-3*β*, a critical downstream element of the Akt pathway, participated in physiological and pathological processes such as regulation of glycogen synthesis and disposal, as well as cell death [32]. It is well accepted that glucose utilization is decreased and FFA oxidation is increased in the diabetic heart [33]. This substrate utilization shift has been known to contribute to the pathogenesis of DCM. Thus, activating Akt phosphorylation and inhibiting GSK-3*β* activity may be considered as cardioprotective actions, as they maintain the physiological growth and functions of the heart and promote cell survival. In the present study, our results indicated that cardiomyocytes exposed to high glucose have strikingly decreased expression levels of Akt and GSK-3*β* phosphorylation, which is in accordance with the previous report [34]. Interestingly, the beneficial roles exerted by curcumin in high glucose-induced cardiac injury such as enhancement of Akt and GSK-3*β* phosphorylation, reduction of gp91^phox^ and p47^phox^ expression, and regulatory apoptosis-related proteins were negated by the application of LY294002, which indicate that the PI3K/Akt/GSK-3*β* signalling pathway may be responsible for the inhibition of high glucose-induced cardiac injury by curcumin.

In summary, curcumin exerts cardioprotection against high glucose-induced cardiomyocyte apoptosis, and these effects were shown to possibly be due to efficient prevention of NADPH oxidase-derived oxidative stress and preservation of Akt and GSK-3*β* phosphorylation in vitro. Therefore, curcumin may be a feasible novel drug for the treatment of DCM.
